# Metabolomics Investigation Reveals Metabolite Mediators Associated with Acute Lung Injury and Repair in a Murine Model of Influenza Pneumonia

**DOI:** 10.1038/srep26076

**Published:** 2016-05-18

**Authors:** Liang Cui, Dahai Zheng, Yie Hou Lee, Tze Khee Chan, Yadunanda Kumar, Wanxing Eugene Ho, Jian Zhu Chen, Steven R. Tannenbaum, Choon Nam Ong

**Affiliations:** 1 Interdisciplinary Research Group in Infectious Diseases, Singapore-MIT Alliance for Research & Technology, Singapore; 2 Department of Pharmacology, Yong Loo Lin School of Medicine, National University Health System, Singapore; 3 Saw Swee Hock School of Public Health, National University of Singapore, Singapore; 4 Koch Institute for Integrative Cancer Research and Departments of Biology, Massachusetts Institute of Technology, Cambridge, Massachusetts, USA; 5 Departments of Biological Engineering and Chemistry, Massachusetts Institute of Technology, Cambridge, Massachusetts, USA; 6 NUS Environment Research Institute, Singapore

## Abstract

Influenza virus infection (IVI) can cause primary viral pneumonia, which may progress to acute lung injury (ALI) and respiratory failure with a potentially fatal outcome. At present, the interactions between host and influenza virus at molecular levels and the underlying mechanisms that give rise to IVI-induced ALI are poorly understood. We conducted a comprehensive mass spectrometry-based metabolic profiling of serum, lung tissue and bronchoalveolar lavage fluid (BALF) from a non-lethal mouse model with influenza A virus at 0, 6, 10, 14, 21 and 28 days post infection (dpi), representing the major stages of IVI. Distinct metabolite signatures were observed in mice sera, lung tissues and BALF, indicating the molecular differences between systematic and localized host responses to IVI. More than 100 differential metabolites were captured in mice sera, lung tissues and BALF, including purines, pyrimidines, acylcarnitines, fatty acids, amino acids, glucocorticoids, sphingolipids, phospholipids, etc. Many of these metabolites belonged to pulmonary surfactants, indicating IVI-induced aberrations of the pulmonary surfactant system might play an important role in the etiology of respiratory failure and repair. Our findings revealed dynamic host responses to IVI and various metabolic pathways linked to disease progression, and provided mechanistic insights into IVI-induced ALI and repair process.

Influenza virus infection (IVI) causes annual epidemics, which result in an estimated 1 billion infections including 3–5 million severe cases and 250,000–500,000 mortality cases worldwide. Two therapeutic strategies, vaccination and antiviral drugs, are currently used to control IVI. However, vaccines need to be updated annually and are effective only if they match with the current circulating virus. Although antiviral drugs may reduce complications of IVI, they need to be given early during infection or used as prophylaxis, and certain strains of influenza viruses can develop resistance to these drugs. As a result, IVI continues to pose a major challenge to the global healthcare systems and there is an urgent need for novel treatment strategies. It has been proposed that host immune responses contributing to severe pathology need to be identified for developing new therapeutics, irrespective of the infecting strains^1^,^2^.

While the most common symptoms of IVI include fever, runny nose, sore throat, cough and fatigue, susceptible populations can have a variety of more severe complications including primary viral pneumonia, which can progress to acute lung injury (ALI) and respiratory failure with a potentially fatal outcome^3^. The molecular mechanisms that give rise to IVI-induced ALI are not well understood, although a prolonged and dysregulated inflammatory response to the infection has been suggested to be the major contributor to severe lung pathology^4^,^5^. Macrophages are recruited to the infected lung and can induce alveolar epithelial cell apoptosis damage and lung injury by expressing tumor necrosis factor-related apoptosis-inducing ligand in severe IVI^6^,^7^. Similarly, neutrophils are also recruited to the inflamed lung during IVI, and although it can limit influenza virus replication^8^, uncontrolled neutrophil activation would disrupt lung homeostasis and contribute to influenza virus pathogenesis by generating excessive reactive oxygen species and releasing harmful granule proteins^9^,^10^. Uncontrolled inflammatory responses can destroy alveoli, induce excessive edema and impairment of alveolar cell function^11^,^12^. Surfactants produced by type II alveolar epithelial cells maintain surface tension at the air–liquid interface, and surfactant degradation brings about high surface tension leading to pulmonary edema, marked impairment of gas exchange and lung mechanical disturbances. Apart from established records of surfactants being comprised of lipids and proteins, details to remaining components are missing^13^. Therefore, despite the importance of IVI-induced aberrations of the pulmonary surfactant system in ALI, it is poorly characterized and the underlying mechanisms still in need of better understanding. Furthermore, given the critical roles played by surfactants in maintaining pulmonary biology^14^, it is tempting to speculate that specific bronchoalveolar lavage fluid (BALF) metabolites may be perturbed and restored over the course of IVI-induced ALI.

Metabolomics, the analysis of the changing metabolite levels in biological systems in response to biological stimuli or perturbations, is a rapidly evolving field in systems biology^15^. As the final downstream products of gene expression, metabolites are directly linked to phenotypes, and reflect cellular activities at the functional level^16^,^17^. Metabolomics and lipidomics has been applied to infectious diseases to study host-pathogen interactions^18^,^19^,^20^,^21^, and recently used to evaluate the efficacy of treatment in IVI-infected mice and influenza patients^22^,^23^. More recently, the micro-RNAs and mRNA expression levels following lung injury and tissue regeneration in a similar model with a different mouse strain were also measured^24^. In our previous study, we systematically characterized cytokines, proteome, and markers of macrophage and neutrophil activities in serum and BALF of a non-lethal murine influenza pneumonia model with infection-induced ALI^25^. During infections, pathogens often exploit host machinery that controls cellular metabolic processes to obtain nutrients and co-factors to proliferate, survive, and avert host immune defense. Conversely, the host regulates the metabolic machinery and the metabolome to defend against the pathogens, shape the immune system and establish repair. At the same time, the metabolome could be representative of tissue injury as a result of exaggerated inflammation. In view of this, we employed global metabolomics analyses of sera, lung tissues and BALF in this established non-lethal influenza murine model, with the aim of identifying key metabolic pathways linked to disease progression and understanding the molecular mechanisms of IVI-induced aberrations of the pulmonary surfactant system during ALI. Herein, we described the dynamics of host responses to IVI, and laid out the temporal changes occurring within the metabolic pathways, observing distinct metabolome changes in serum, lung and BALF indicative of the differences between systematic and localized host responses to IVI. Importantly, we found perturbations and restoration of pulmonary surfactant phospholipids and other metabolites in different phases of IVI in an ordered chronological sequence. The mechanisms leading to these metabolic changes were also explored.

## Results

### Temporal pathophysiologic profile in the non-lethal mouse model of IVI

**Figure 1:**
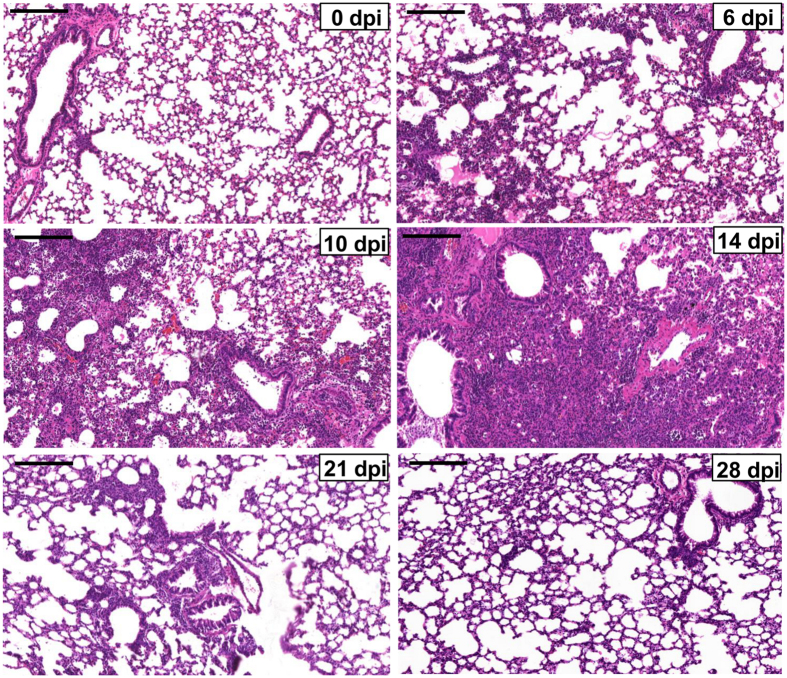
Representative images of H&E staining of lung tissue sections from mice infected with sub-lethal PR8 influenza A virus. Representative H&E images of lung sections showing tissue damage progression at the indicated time points (dpi) after influenza A infection. Scale bar = 100 μm. Each picture is representative for 8 mice.

We first established the temporal pathophysiologic profiles of the mouse model in response to IVI and the results were consistent with previous characterization of the same model^25^,^26^,^27^,^28^. Viral titers rose quickly and peaked at about 5 to 6 dpi, which then declined and virtually disappeared by 13 to 14 dpi. The body weight loss of the mice started at about 5 dpi and reached the lowest weight at around 9 to 10 dpi. IVI induces immune responses in the mice and leads to immune cell infiltration into the lung. Infiltration was detected at 5 to 7 dpi and increased continuously until reaching the peak level at 14 dpi, when most of viruses were cleared (Fig. 1). Thereafter, tissue repair and regeneration began to restore pulmonary homeostasis. By 21 and 28 dpi, the infiltration was significantly reduced, a reflection of lung tissue repair and recovery of the mice. Based on these model characteristics, serum, lung tissue and BALF samples were correspondingly collected at 0, 6, 10, 14, 21 and 28 dpi for the metabolomics study, representing major stages associated with peak viremia, body weight loss, inflammatory lung injury, and recovery phase of the infection.

### Metabolic profiles of mice sera, BALF and lung tissues

In order to obtain reliable metabolic profiles of the samples, it is important to ensure the robustness of the analytical method. We first evaluated the stability and reproducibility of the LC-MS method by performing PCA on all the samples including the 6 QC samples^29^. As shown in Fig. S1, the QC samples are clustered in PCA scores plots of sera, lung tissues and BALF (Supp Fig. S1A–C), indicating good stability and reproducibility of the chromatographic separation during the whole sequence. Next, PCA scores plots were used to study metabolic profiles of the samples, and distinct temporal profiles of changes were observed in mice sera, lung tissues and BALF metabolomes upon IVI. PCA scores plot showed that metabolome changes in serum were reversible and the most predominant changes, as compared with controls at 0 dpi, happened at 6 dpi, indicated by the furthest cluster of mice at 6 dpi from the cluster at 0 dpi (Supp Fig. S1A). At 10 dpi, the changes moved back slightly, though its clustering was scattered and some data points (mice) moved closer to the cluster at 0 dpi. The metabolome changes returned to near control levels at 14 dpi, revealed by the close clusters of 14 and 0 dpi, and kept at control levels at 21 and 28 dpi. In PCA scores plot of lung tissues, a clear time-course of metabolome changes could be observed. The changes started at 6 dpi, which increased continuously at 10 dpi and peaked at 14 dpi, and then moved towards the control levels at 21 and 28 dpi (Supp Fig. S1B). Unlike reversible changes in serum, certain metabolome changes in lung did not return to the control levels even at 28 dpi. Reversible metabolome changes in BALF were also observed in PCA scores plot, and the most predominant changes happened at 10 dpi (Supp Fig. S1C).

### Identification of significantly altered metabolites and pathways

To determine significantly differential metabolites, metabolites were filtered based on the following criteria: (i) *p* < 0.05 in one-way ANOVA with Benjamini-Hochberg Multiple Testing Correction; (ii) FC ≥ 1.5; (iii) signal intensity ≥20,000 counts (approximately three times the limit of detection of our LC/MS instrument)^30^. The structure identification of the differential metabolites was based on our published work (19), and the identification process is illustrated here with cyclic adenosine monophosphate (cAMP). First, the element composition C_10_H_12_N_5_O_6_P of the *m/z* 329.05 ion was calculated based on the exact mass, the nitrogen rule and the isotope pattern by Masshunter software from Agilent (Supp Fig. S2A). Then, the elemental composition and exact mass were used for open source database searching, including HMDB (http://www.hmdb.ca/), METLIN (http://metlin.scripps.edu/) and MassBank (http://www.massbank.jp/). Next, MS/MS experiments were performed to obtain structural information via the interpretation of the fragmentation pattern of the metabolite, and the characteristic fragment ion of adenosine base was clearly observed in both positive (*m/z* 136.06) and negative (*m/z* 134.04) MS/MS spectra (Supp Fig. S2B). The MS/MS spectra of possible metabolite candidates in the databases were also searched and compared. As a result, the metabolite was identified as cAMP, which was finally confirmed by comparing both RT in the LC and MS/MS spectra with those of the standard (Supp Fig. S2C).

**Figure 2:**
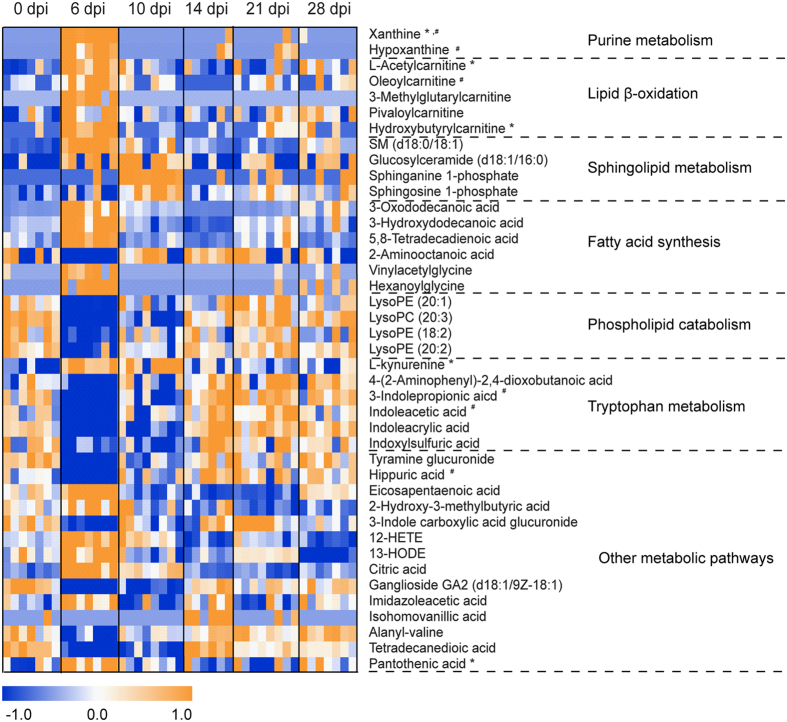
Heat map of identified differential metabolites in serum. Each row shows ion intensity for a specific metabolite after mean centering and unit variance scaling of the data. Each column shows the serum metabolic profiles of PR8-infected mice at 0 dpi, 6 dpi, 10 dpi, 14 dpi, 21 dpi, and 28 dpi. Regions of red or blue indicate that the metabolite content is increased or decreased, respectively. *Common differential metabolites in the lung. ^#^Common differential metabolites in bronchoalveolar lavage fluid.

**Figure 3:**
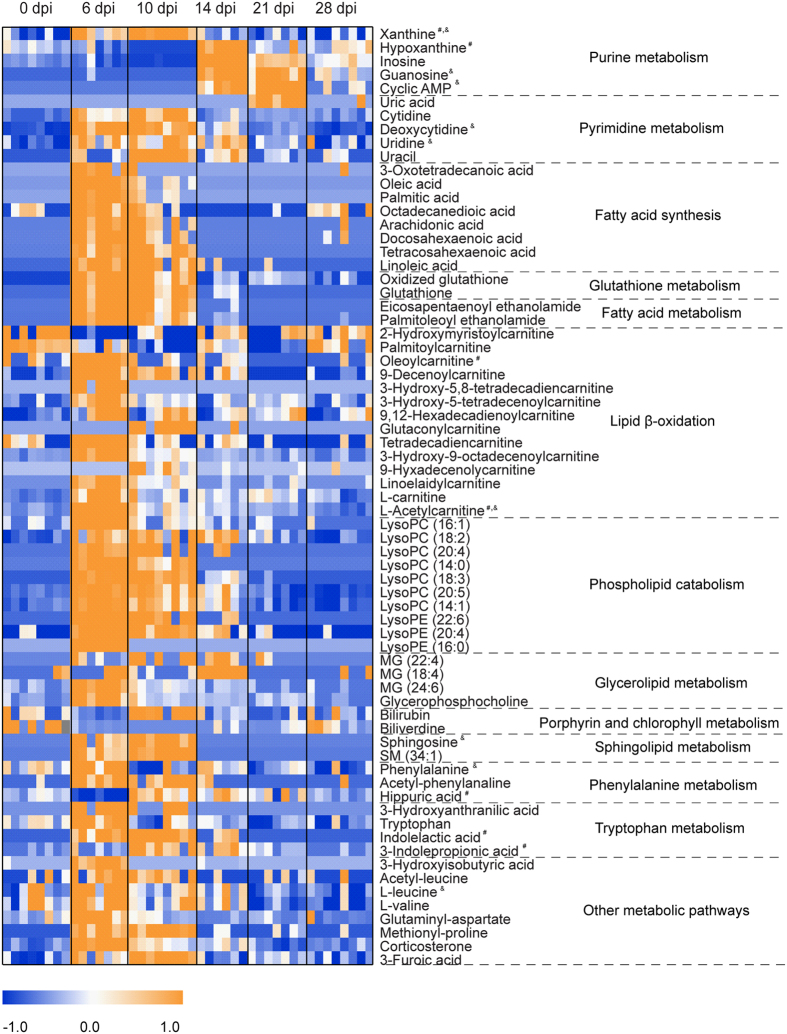
Heat map of identified differential metabolites in bronchoalveolar lavage fluid. Each row shows ion intensity for a specific metabolite after mean centering and unit variance scaling of the data. Each column shows the serum metabolic profiles of PR8-infected mice at 0 dpi, 6 dpi, 10 dpi, 14 dpi, 21 dpi, and 28 dpi. Regions of red or blue indicate that the metabolite content is increased or decreased, respectively. ^#^Common differential metabolites in serum. ^&^Common differential metabolites in the lung.

**Figure 4:**
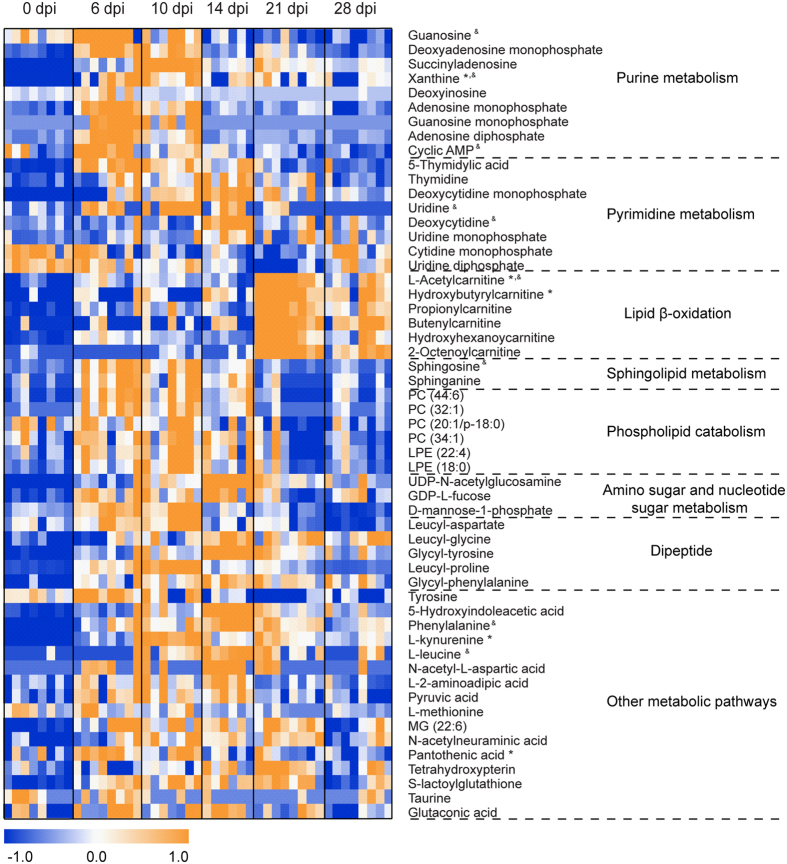
Heat map of identified differential metabolites in the lung. Each row shows ion intensity for a specific metabolite after mean centering and unit variance scaling of the data. Each column shows the serum metabolic profiles of PR8-infected mice at 0 dpi, 6 dpi, 10 dpi, 14 dpi, 21 dpi, and 28 dpi. Regions of red or blue indicate that the metabolite content is increased or decreased, respectively. *Common differential metabolites in serum. ^&^Common differential metabolites in bronchoalveolar lavage fluid.

With above-mentioned method, 41, 55 and 69 differential metabolites were identified in sera, lung tissues and BALF, respectively, of which they belonged to a variety of metabolite classes (Figs 2, 3, 4, Supp Table S1). In serum, the major metabolite classes are acylcarnitine, fatty acid, amino acid and derivatives, sphingolipid, phospholipid and purine. In lung and BALF, although main metabolite classes are similar to those in the serum, the identities and the number of metabolites in each class are quite different. For example, a total of 26 purines and pyrimidines were found in lung and BALF, including purine and pyrimidine bases and derivatives, and nucleotides with one or two phosphate groups, while only 2 purines were found in serum. Similar to PCA scores plots, the heatmaps of altered metabolites showed that the changes of these metabolites were reversible in serum and BALF, with the most significant changes happening at 6 and 10 dpi, respectively (Figs 2 and 3). Although most of the identified differential lung metabolites also showed reversibility in their changes (Fig. 4), certain metabolites, especially acylcarnitines, still remained at an elevated level at 28 dpi. In terms of global quantitative differences, five differential metabolites were commonly identified between serum and lung, seven were common between serum and BALF, and eight were common between BALF and lung. Additionally, a group of amino acids and nucleotide sugars were uniquely identified in the lung.

**Figure 5:**
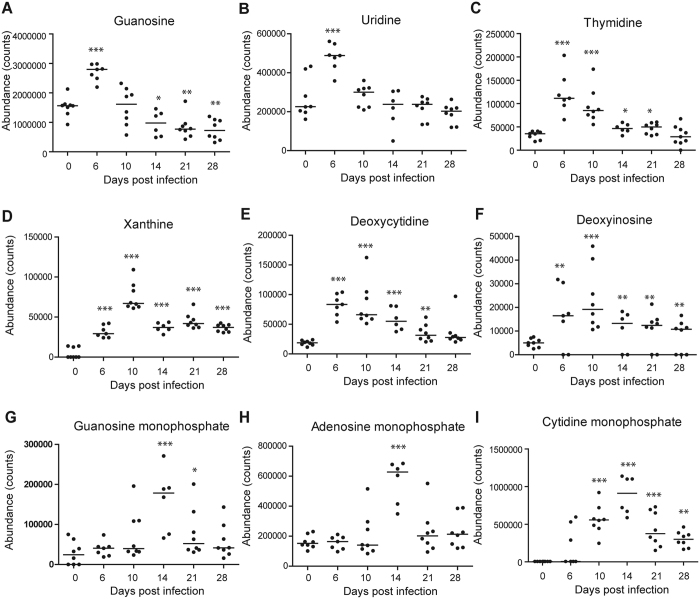
Scatter plots of nine significantly altered purines and pyrimidines in the lung. (**A**) Guanosine (**B**) Uridine (**C**) Thymidine (**D**) Xanthine (**E**) Deoxycytidine (**F**) Deoxyinosine (**G**) Guanosine monophosphate (**H**) Adenosine monophosphate (**I**) Cytidine monophosphate. Horizontal lines represent median value. *p < 0.05, **p < 0.01, ***p < 0.001, by one-way ANOVA test. The statistical comparison was with control levels.

**Figure 6:**
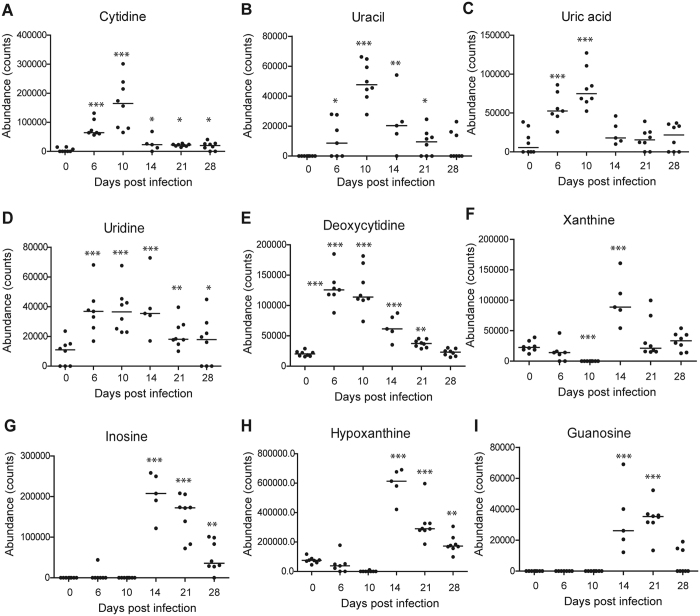
Scatter plots of nine significantly altered purines and pyrimidines in bronchoalveolar lavage fluid. (**A**) Cytidine (**B**) Uracil (**C**) Uric acid (**D**) Uridine (**E**) Deoxycytidine (**F**) Xanthine (**G**) Inosine (**H**) Hypoxanthine (**I**) Guanosine. Horizontal lines represent median value. *p < 0.05, **p < 0.01, ***p < 0.001, by one-way ANOVA test. The statistical comparison was with control levels.

Two pathway analysis tools, MetaboAnalyst and IPA were used to identify altered pathways related to IVI, and both tools provided similar results (Supp Fig. S3A,B). In MetaboAnalyst, the pathway data are derived from the KEGG database (www.genome.jp/kegg/). In the overview graph of pathway analysis with MetabolAnalyst (Supp Fig. S3A), all the matched pathways were arranged by −log(p) values from pathway enrichment analysis on Y-axis and pathway impact values from pathway topology analysis, which uses node centrality measures to estimate node importance on X-axis. The node color is based on its p value and the node radius is determined based on their pathway impact values^31^. In the bar chart of altered pathways with IPA, the Y-axis was the −log(p) values of each pathway calculated using right-tailed Fisher’s exact test (Supp Fig. S3B). In serum, major perturbed metabolic pathways included tryptophan metabolism, fatty acid *β*-oxidation, sphingolipid metabolism, purine metabolism, pantothenate and CoA biosynthesis, histamine degradation, etc (Supp Fig. S4). In BALF and lung, major altered metabolic pathways were purine and pyrimidine metabolism, sphingolipid metabolism, glutathione metabolism, fatty acid biosynthesis, fatty acid *β*-oxidation, arachidonic acid metabolism, and so on (Figs 5 and 6, Supp Figs S3 and S5). Disturbed amino sugar and nucleotide sugar metabolism pathways were uniquely identified in the lung. The molecular and cellular functions of BALF and lung with IPA were mainly cellular growth and proliferation, cellular development, free radical scavenging, DNA replication, and so on (Supp Fig. S3C), which are consistent with the functions identified in transcriptomics study of the model^24^.

### Dysregulated pulmonary surfactants phospholipid hydrolysis following IVI

**Figure 7:**
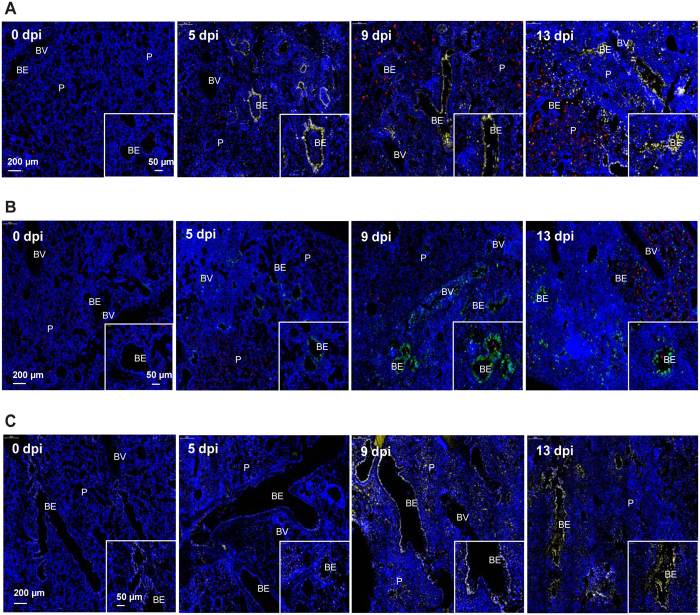
Degradation and regulation of pulmonary surfactants during sub-lethal PR8 influenza infection. Representative immunofluorescence staining of secretory phospholipase A_2_ (sPLA_2_) (**A**) and its activated form, sPLA_2_^p505^ (**B**) to demonstrate surfactant PC hydrolysis to LPC progression at the indicated time points after influenza infection on mice. (**C**) Representative immunofluorescence staining of 11β-HSD on mouse lung sections from PR8-infected mice to demonstrate increased corticosterone production in bronchial epithelium. Immune cell infiltration can be observed at regions with dense DAPI stains. Scale bar = 200 μm. Insert scale bar = 50 μm. Each image is representative of 3–5 mice. BV = Blood vessel; BE = Bronchiole; P = Parenchyma.

Surfactants are critical to pulmonary biology and influenza infections result in surfactant function impairment, leading to alveoli collapse and ALI^14^. Therefore, we focused on understanding the impact of BALF metabolome with IVI and their enzymatic regulation at the major metabolome shifts at 5 to 14 dpi. Mouse lung tissues for immunofluorescence analysis were collected at 0, 5, 9 and 13 dpi to capture enzymatic reactions potentially preceding the metabolism and catabolism of the metabolites. We observed significant increase of BALF LPCs and free fatty acids (palmitic, oleic and linoleic acids) at 6 and 10 dpi (Fig. 3) before a steady decrease following 14 dpi. The concomitant increase in sPLA_2_ and its activated form sPLA_2_^pS505^, the enzyme that hydrolyzes the *sn*-2 ester bond of phospholipids, in bronchial epithelial cells, endothelial cells as well as cells in the lung parenchyma and alveolar space beginning from 5 dpi, up to 9 dpi and decreasing from 13 dpi, suggested that the enzyme could account for the increase in LPCs (Fig. 7A,B). A limited but similar set of lyso-phospholipids in the serum showed an opposite trend to that of BALF and tissues, reflecting their crucial functions in immunity and airway injury. The converse is true at 9/10 and 13/14 dpi as viremia recedes. LPCs induce chemotaxis of monocytes^32^,^33^, which matched with the immunofluorescence distribution of sPLA_2_ and sPLA_2_^pS505^ in regions heavily infiltrated by immune cells, as shown by dense DAPI staining at 9 and 13 dpi (Fig. 7A,B). We also observed increased perilipin immunostaining in the lipofibroblasts (Supp Fig. S6), increased tissue PCs peaking at 10 dpi (Fig. 4) and BALF MGs from 6 dpi to 14 dpi (Fig. 3), suggesting compensatory measures to restore surfactant phospholipids. At 9 and 13 dpi, upregulation of SP-A, a small hydrophobic protein of 29–36 kDa that enhances the surface tension lowering properties of surfactant phospholipids was observed. This coincided with an increase in corticosterone levels and upregulation of its enzyme, 11-beta-steroid dehydrogenase (11β-HSD) which stimulates SP-A expression^34^ (Fig. 7C). There was a dramatic increase in aggregated SP-A levels in alveolar space and in ring-like structures surrounding several alveoli at 14 dpi, plausibly a result of SP-A neutralizing and inhibiting PR8 virus^35^,^36^ and interaction with the other components of surfactant system. Moreover, the decrease in sPLA_2_ and sPLA_2_^pS505^ immunostaining at 13 dpi coincided with a sharp increase in SP-A, plausibly reflecting the sPLA_2_ inhibitory effect by SP-A^37^,^38^. Taken together, the metabolome and companion protein data suggests IVI induced early damage of surfactants but restoration of surfactant phospholipids in combination with SP-A to maintain surfactant integrity at 13 dpi as the virus was cleared.

## Discussion

ALI is a severe complication of primary influenza viral pneumonia, adversely affecting the pulmonary surfactant system; yet the underlying mechanisms are not well understood and currently there are still no pathophysiologic-driven therapeutic strategies available. We conducted metabolomics study on an established non-lethal mouse model of influenza pneumonia with the aim of unraveling the pathogenesis of IVI-induced ALI. In this model, inflammatory damage of the lung started at 5 to 7 dpi and peaked at 14 dpi, a time point when viral load has subsided, which strongly indicates that host factors would contribute to lung injury. In order to capture the dynamic metabolic responses to IVI, BALF, together with lung tissue and serum were profiled at 0, 6, 10, 14, 21 and 28 dpi, covering the major stages of ‘maximal viremia’, ‘peak body weight loss’, ‘maximal inflammatory lung damage’, as well as ‘recovery phase’ of the infection. For immunofluorescence analysis, lung tissues were collected at 0, 5, 9 and 13 dpi to capture enzymatic reactions potentially preceding the metabolism and catabolism of the metabolites. Compared to our previous analyses of cytokines, proteome and protein adducts changes in serum and BALF of the same mouse model^25^, lung tissue as well as serum and BALF at two more sampling time points, 10 and 28 dpi, were included in the current study to provide a more comprehensive metabolite kinetics profile of IVI-induced ALI. Importantly, this approach offered novel insights into the pathological restoration process of the surfactant metabolome and allowed us to relate and verify changes to specific metabolite levels to their respective proteins.

Pulmonary surfactants are critical to pulmonary biology, consist mainly of lipids and a smaller fraction of proteins, are synthesized by type II alveolar epithelial cells and lower pulmonary surface tension for efficient gaseous exchange. Our metabolomics analysis revealed marked differences in BALF metabolome at 6, 10 and 14 dpi following PR8 inoculation. As a consequence of upregulation and activation of sPLA_2_ at 5 and 9 dpi, we observed a corresponding enrichment of LPCs and fatty acids at 6 and 10 dpi before declining at 14 dpi (Fig. 3). As confirmed by immunofluorescence, we did not expect sPLA_2_ to co-localize with type II alveolar epithelial cells where surfactants were synthesized, but instead were expressed largely in the bronchioles (Fig. 7A,B). Phospholipid analysis of lung surfactant PCs using GC-MS showed that ~60% of PCs at physiological states are predominantly dipalmitoylphosphatidylcholine (PC 16:0/16:0), and the rest made up of 16:0/14:0, 16:0/16:1, 16:0/18:1 and 16:0/18:2 PC molecular species^39^. Interestingly, the acyl chains of many LPCs (14:0, 14:1, 16:1, 18:2) and fatty acids (palmitic, oleic acids) found in this study were congruent with the composition of lung surfactant PC species^13^,^39^. Thus we can conclude that the increase in LPCs and fatty acids is the result of PC hydrolysis by sPLA_2_. LPCs play crucial roles in ALI by affecting airway and capillary permeability^40^, increasing cellular permeability of alveolar type I epithelial cells^41^, inducing immune cells chemotaxis^32^,^33^ and decreased lung compliance and ventilator mechanisms^42^. Subsequent restoration of surfactant function and structural mechanisms including surfactant PCs is required for efficient gaseous exchange. Indeed, several lines of evidence strongly suggest the restoration of the surfactant system starting from 13/14 dpi. The attenuation of sPLA_2_ activity by SP-A^37,38^, the latter plausibly brought about by the induction of 11β-HSD which catalyzes the formation of BALF corticosterone, which led to decreased BALF LPCs; and increased BALF MGs and upregulation of perlipin A in lipofibroblasts to support triacylglycerols metabolism for the synthesis of surfactant PCs^43^, are indications of surfactant system recovery (Figs 3 and 7C, Supp Fig. S4). Our spatiotemporal results further demonstrate how the different lung cell types and compartments interact in a dynamic fashion to return surfactants to homeostatic levels.

In addition to these findings, our metabolomics analysis also uncovered novel metabolites which were associated with surfactants. Purines and pyrimidines are important signalling molecules and 26 differential purines and pyrimidines were identified in lung and BALF after IVI. In signalling, purines and pyrimidines exert their modulating effects mainly through purinergic signalling receptors, which have been implicated in lung injury and in the pathogenesis of a variety of respiratory diseases^44^. Activated P2Y receptors could upregulate inducible nitric-oxide synthase expression in lung immune cells, resulting in increased reactive oxygen species (ROS) and reactive nitrogen species (RNS) mediated stress in the lung^45^,^46^. Excessive production of ROS and RNS could attack cellular molecules like DNA, proteins and lipids to induce cellular and tissue damage, and has been implicated in IVI-induced ALI^47^,^48^. Indeed, elevated levels of nitrosative deamination products of DNA, including uracil, hypoxanthine, inosine and xanthine, were found in both lung tissue and BALF during the infiltration of immune cells in our study. Furthermore, glutathione, a key antioxidant which protects cellular macromolecules against ROS and RNS in lung airways^49^,^50^, decreased dramatically at 6 dpi in BALF and did not start recovering until 28 dpi. Conversely, the oxidized form of glutathione that sequesters excess ROS, peaked at 10 dpi (Fig. 3). We have previously shown that the level of 3-chlorotyrosine, a protein adduct formed from interaction with nitric oxide and an indicator of neutrophil activity, increased and peaked at 14 dpi in serum of the same model upon IVI^25^. It had also been shown that key lung-abundant antioxidant enzymes of the model could be depleted by IVI^28^. Collectively, our metabolome results significantly add to the multicomponent composition of surfactants^13^.

Compared to the controls, 24 out of the 26 identified purines and pyrimidines were elevated and peaked between 6 to 14 dpi. Increased release of purine nucleotides from epithelial and immune cells in the airway has been observed during inflammation, and is believed to play an important role in the pathophysiology of chronic lung diseases^50^. Among these purines and pyrimidines, uridine diphosphate (UDP), peaked at 10 dpi in our study, could stimulate the release of antimicrobial chemokine ligand 20 and regulate immune cells recruitment in inflamed airways by acting on both epithelial and immune cells^52^. Meanwhile, adenosine monophosphate (AMP), which peaked at 14 dpi in our study, is a candidate biomarker of airway inflammation and the measurement of AMP in sputum was proposed as a non-invasive method to track this inflammation^53^. It is noteworthy that unlike most purines and pyrimidines, cAMP showed a decreased change trend and reached the lowest level at 21 dpi in the lung. cAMP is an important second messenger and its generation and inhibition are well-controlled by extracellular first messengers, including neurotransmitters, hormones, chemokines or prostaglandins^54^. It regulates a broad range of cellular processes, including immune functions, and increased intracellular cAMP suppresses immune functions by decreasing inflammatory mediator generation, phagocytosis and the killing of microbes^55^. Thus, decreased cAMP level in the lung in our study might contribute to ALI by stimulating inflammation. In fact, it has been proposed that drugs which could increase intracellular cAMP levels may be useful to treat chemically induced ALI by stimulating alveolar fluid transport, inhibiting inflammation and inducing bronchodilation^56^.

Distinct metabolic signatures of serum, BALF and lung were observed in both PCA scores plots and heatmaps of the differential metabolites, with peaked metabolome changes happening at 6, 10 and 14 dpi, respectively. The metabolome changes in serum represent systemic host responses to the infection, and although metabolic profile of serum generally coincided with the kinetics of viral replication and clearance, our results showed that such changes did not reflect the progress of lung pathology. In fact, when lung injury reached the maximal level at 14 dpi, the metabolome changes in serum had returned to near control levels (Fig. 2). Although systemic metabolome changes could potentially reflect processes happening in all tissues and organs, including tissue lesions, organ dysfunctions, etc., it has been found that the analysis of serum/plasma is not adequate to assess the metabolic changes in tissues and even dramatic metabolic changes in the tissues are not necessarily observed in the plasma^57^. Conversely, the metabolome changes in lung, which represent localized host responses to IVI, reached the maximal levels at 14 dpi and were consistent with the progress of lung pathology. Meanwhile, the metabolome changes in BALF coincided with the time course of weight loss of the mice and peaked at around 10 dpi. These results indicate that metabolome changes in different type of samples could provide different information concerning disease progression. Another metabolic profile difference between serum/BALF and lung is that both PCA scores plots and heatmaps revealed reversible metabolome changes in serum and BALF upon IVI, which are consistent with the sub-lethal model and the recovery of the mice. On the other hand, metabolome changes in the lung did not return to the control levels even at 28 dpi, indicating that IVI resulted in a more prolonged metabolic effect in the infected airways. Based on these observations, we concluded that parallel metabolic profiling of multiple biofluids (serum and BALF) and lung tissues in our study is necessary to capture more comprehensive metabolic changes of the mice which may directly relate to ALI.

In conclusion, this study is the first in providing a comprehensive description of metabolome changes in a mouse model of influenza pneumonia in the bronchoalveolar lavage fluid, lung and serum, tracing along the major stages of IVI-induced ALI. Through our approach we were able to offers mechanistic and spatiotemporal insights into IVI-induced surfactant disturbances and how the host responds subsequently for repair and restoration. In addition, other altered metabolic pathways and molecules may serve as disease-relevant targets for designing pathophysiologic-driven therapeutics to alleviate severe IVI.

## Methods

### Mouse and influenza virus

The non-lethal mouse model has been well-established^25^,^26^,^27^,^28^. Briefly, C57BL/6 (B6) mice at 8–12 weeks of age were purchased from the Centre for Animal Resources (CARE), Singapore. Influenza A/Puerto Rico/8/34 H1N1 virus (PR8) was provided by Professor Vincent TK Chow (National University of Singapore, Singapore). Mice were infected with a sub-lethal dose of influenza virus by intra-tracheal administration after anesthetization. At 0, 6, 10, 14, 21 and 28 day post infection (dpi), 8 mice of each time point were euthanized and sera, BALF and lung tissues were collected, as indicated in previous publications^25^,^26^,^27^,^28^.

This study was carried out in strict accordance with the National Advisory Committee for Laboratory Animal Research (NACLAR) Guidelines (Guidelines on the Care and Use of Animals for Scientific Purposes) in facilities licensed by the Agri-Food and Veterinary Authority of Singapore (AVA), the regulatory body of the Singapore Animals and Birds Act. The protocol was approved by the Institutional Animal Care and Use Committee (IACUC), National University of Singapore. Mice were monitored every day after infection, and any mouse with 30% body weight loss will be euthanatized immediately.

### Sample preparation

The procedure for serum and BALF sample preparation followed our previously published report^19^. Briefly, a volume of 50 μL serum or 100 μL BALF was thawed at 4 °C and proteins were precipitated with 200 or 400 μL ice-cold methanol, which contained 5 μg/mL 9-fluorenylmethoxycarbonyl-glycine as an internal standard. After vortexing, the mixture was centrifuged at 17,000 *g* for 10 minutes at 4 °C and the supernatant was collected and evaporated to dryness in a vacuum concentrator. The dry extracts were then resuspended in 100 μL of 98:2 water/methanol for liquid chromatography/mass spectrometry (LC-MS) analysis. Quality control (QC) samples were prepared by mixing equal amounts of serum or BALF from all the samples and processed as real samples. All samples were kept at 4 °C and analyzed in a random manner. The QC sample was run after each 8 samples to monitor the stability of the system.

The procedure for lung tissue preparation followed published report with some modifications^58^. Briefly, a 5 mg section of lung tissue was placed in bead-beater tube and homogenized with 100 μL silica bead in 700 μL of prechilled methanol/water (1:1) using Qiagen TissueLyser. The mixture was centrifuged at 17,000 *g* for 10 min at 4 °C and the supernatant was transferred to an Eppendorf tube. Then 700 μL of prechilled methanol/water (1:1) was added to the pellet and vortexed. The mixture was again centrifuged at 17,000 *g* for 10 min at 4 °C and the supernatant was combined with the supernatant from previous step. The mixed supernatant was lyophilized to dryness in a vacuum concentrator. The dry extracts were resuspended in 200 μL of 98:2 water/methanol for LC-MS analysis, and 20 μL from each sample was pooled as QC samples. All samples were kept at 4 °C and analyzed in a random manner. The QC sample was run after each 8 samples to monitor the stability of the system.

### Metabolomics analysis by UPLC-QTOFMS

The metabolomics analysis followed our previously published report with some modifications^19^. Briefly, reversed-phase liquid chromatography (RPLC)-MS analyses were performed using Agilent 1290 ultrahigh pressure liquid chromatography system (Waldbronn, Germany) equipped with a 6520 Q-TOF mass detector managed by a MassHunter workstation. The column used for the separation was rapid resolution HT Zorbax SB-C18 (2.1 × 100 mm, 1.8 μm; Agilent Technologies, USA), and the mobile phase was (A) 0.1% formic acid in water and (B) 0.1% formic acid in methanol. The initial condition of the gradient elution was set at 2% B for 2 min with a flow rate of 0.4 ml/min. A 7 min linear gradient to 70% B was then applied, followed by a 5 min gradient to 100% B which was held for 3 min. The sample injection volume was 10 μL and the oven temperature was set at 40 °C.

The electrospray ionization mass spectra were acquired in both positive and negative ion mode. Mass data were collected between *m/z* 100 and 1000 at a rate of two scans per second. The ion spray voltage was set at 4,000 V for positive mode and 3,500 V for negative mode. The heated capillary temperature was maintained at 350 °C. The drying gas and nebulizer nitrogen gas flow rates were 12.0 L/min and 50 psi, respectively. Two reference masses were continuously infused to the system to allow constant mass correction during the run: *m/z* 121.0509 (C_5_H_4_N_4_) and *m/z* 922.0098 (C_18_H_18_O_6_N_3_P_3_F_24_).

### Data analysis and compound identification

Raw spectrometric data were analyzed by MassHunter Qualitative Analysis software (Agilent Technologies, US) and the molecular features, characterized by retention time (RT), chromatographic peak intensity and accurate mass, were obtained by using the Molecular Feature Extractor algorithm. The features were then analyzed by MassHunter Mass Profiler Professional software (Agilent Technologies, US). Only features with an intensity ≥20,000 counts (approximately three times the limit of detection of our LC/MS instrument) and found in at least 80% of the samples at the same sampling time point signal were kept for further processed. Next, a tolerance window of 0.15 min and 2 mDa was used for alignment of RT and *m/z* values, and the data were also normalized by internal standard. For statistical analysis, one-way ANOVA (*p* < 0.05) with Benjamini-Hochberg Multiple Testing Correction was employed. The fold change (FC) analysis was also performed between the group at each of the other time points relative to the control group to further filter the features and only those features with FC > 1.5 were selected as potential significantly altered metabolites. Unsupervised multivariate analysis principal components analysis (PCA) was performed with the Unscrambler-X statistical software package (CAMO software, Oslo, Norway). Supervised multivariate analysis orthogonal partial least-squares discriminant analysis (OPLS-DA) was performed with the SIMCA-P software (Umetrics AB, Umea, Sweden). The features with Variable Importance in the Projection (VIP) values greater than 1 were considered to be influential for the separation of samples in OPLS-DA analysis.

The structure identification of the differential metabolites was based on our published work^19^. Briefly, the element compositions of the metabolites were first calculated based on the exact mass, the nitrogen rule and the isotope pattern by Masshunter software from Agilent. Then, the elemental composition and exact mass were used for open source database searching, including LIPIDMAPS (http://www.lipidmaps.org/), HMDB (http://www.hmdb.ca/), METLIN (http://metlin.scripps.edu/) and MassBank (http://www.massbank.jp/). Next, MS/MS experiments were performed to obtain structural information via the interpretation of the fragmentation pattern of the metabolite. The MS/MS spectra of possible metabolite candidates in the databases were also searched and compared. Finally, the metabolites were confirmed by comparison with the standards when they are commercially available. For metabolic pathway analysis, Ingenuity Pathway Analysis software (IPA, www.ingenuity.com) and metaboAnalyst^59^ were used to identify relevant pathways.

### Immunofluorescence staining

Mouse lung tissues were collected at 0, 5, 9 and 13 dpi, and processed as previously described^60^. Immunofluorescence staining was performed according to our previous published report^28^. Dissected lung were fixed in 10% formalin for at least 48 h and processed using Leica TP-1050 tissue processor (Leica Instruments; Oberkochen, Germany) and embedded in paraffin wax. 5 μm-thick lung sections were cut, dewaxed, rehydrated in 100%, 90% and 70% absolute ethanol. Target antigen retrieval was performed using citrate buffer pH 6.0 (Dako Corporation, Carpinteria, CA). Buffer was pre-heated using medium high power microwave for 5 min and slides were immersed in sub-boiling antigen retrieval buffer for 20 min in low power microwave. After washing, slides were blocked with 10% serum (same host as secondary antibody) for 1 h at room temperature. This is followed by incubation with primary antibody in closed humidified chamber overnight at 4 °C. After washing, slides were incubated with secondary antibody protected from light, at room temperature for 1 h, followed by 4’,6 diamidino-2-phenylindole (DAPI) staining and mounted in SlowFade Gold anti-fade reagents (Life Technologies, Gaithersburg, MD). Lung sections were immunofluorescence stained for secretory phospholipase A_2_ (sPLA_2_, ab58375), secretory phospholipase A_2_ (sPLA_2_^phosphoS505^; ab53105), Perilipin A (ab3526) and hydroxysteroid (11-beta) dehydrogenase 1 (HSD11B1, ab39364) (Abcam, Cambridge, MA). All sections were image at 5×, 10× and 40× with Mirax Midi slide scanner (Carl Ziess).

## Additional Information

**How to cite this article**: Cui, L. *et al.* Metabolomics Investigation Reveals Metabolite Mediators Associated with Acute Lung Injury and Repair in a Murine Model of Influenza Pneumonia. *Sci. Rep.* **6**, 26076; doi: 10.1038/srep26076 (2016).

## Supplementary Material

Supplementary Information

Supplementary data 1

Supplementary data 2
