# Supplementary material for: Metabolomics Investigation Reveals Metabolite Mediators Associated with Acute Lung Injury and Repair in a Murine Model of Influenza Pneumonia
Source: Sci Rep. 2016 May 18;6:26076. doi: 10.1038/srep26076 (PMC4870563; doi:10.1038/srep26076)

Metabolomics Investigation Reveals Metabolite Mediators Associated with Acute Lung Injury and Repair in a Murine Model of Influenza Pneumonia

Liang Cui[1¶], Dahai Zheng[1¶], Yie Hou Lee[1¶†], Tze Khee Chan[1,2], Yadunanda Kumar[1¤], Wanxing Eugene Ho[3], Jian Zhu Chen[1,4], Steven R. Tannenbaum[1,5*], Choon Nam Ong[1,3,6*],

Figure S1. PCA score plots of PR8-infected mice sera, lung tissues and bronchoalveolar lavage fluid. A. serum. **B.** lung. **C.** bronchoalveolar lavage fluid (BALF). The principal component analysis (PCA) model was constructed using combined LC-MS metabolomics data from mice at 0 dpi (D0), 6 dpi (D6), 10 dpi (D10), 14 dpi (D14), 21 dpi (D21), and 28 dpi (D28).

Figure S2. Identification of cyclic adenosine monophosphate (cAMP) as a differential metabolite in the lung. A. the extracted ion chromatogram (EIC) and matched formula of the ion *m/z* 329.05. **B.** MS/MS spectrum in both positive and negative modes and proposed fragmentation of the ion *m/z* 329.05. **C.** EIC and MS/MS spectrum in both positive and negative modes of a commercial standard cAMP.

Figure S3. Pathways analysis with Ingenuity Pathway Analysis and Metaboanalyst based on differential metabolites in the lung. A. altered pathways with Metaboanalyst. The matched pathways were arranged by –log(p) values from pathway enrichment analysis on Y-axis and pathway impact values from pathway topology analysis, which uses node centrality measures to estimate node importance, on X-axis. The node color is based on its p value and the node radius is determined based on their pathway impact values. **B.** the bar chart of the top 20 altered pathways with Ingenuity Pathway Analysis. The Y-axis was the –log(p) values of each pathway calculated using right-tailed Fisher's exact test. **C.** top five molecular and cellular functions revealed by Ingenuity Pathway Analysis.

Figure S4. Pathways analysis with Ingenuity Pathway Analysis and Metaboanalyst based on differential metabolites in serum. A. altered pathways with Metaboanalyst. The matched pathways were arranged by –log(p) values from pathway enrichment analysis on Y-

axis and pathway impact values from pathway topology analysis, which uses node centrality measures to estimate node importance, on X-axis. The node color is based on its p value and the node radius is determined based on their pathway impact values. **B.** the bar chart of the top 20 altered pathways with Ingenuity Pathway Analysis. The Y-axis was the –log(p) values of each pathway calculated using right-tailed Fisher's exact test.

Figure S5. Pathways analysis with IPA and Metaboanalyst based on differential metabolites in BALF. A. altered pathways with Metaboanalyst. The matched pathways were arranged by –log(p) values from pathway enrichment analysis on Y-axis and pathway impact values from pathway topology analysis, which uses node centrality measures to estimate node importance, on X-axis. The node color is based on its p value and the node radius is determined based on their pathway impact values. **B.** the bar chart of the top 20 altered pathways with Ingenuity Pathway Analysis. The Y-axis was the –log(p) values of each pathway calculated using right-tailed Fisher's exact test. **C.** top five molecular and cellular functions revealed by Ingenuity Pathway Analysis.

Figure S6. Restoration of surfactant phospholipid synthesis by perlipin A. Representative immunofluorescence staining of perilipin A on mouse lung sections from PR8-infected mice suggesting increased phospholipid production as a result of triacylglycerol metabolism. Scale bar = 200 μm. Insert scale bar = 50 μm. Each image is representative of 3-5 mice.

Table S1. Identified differential metabolites in lung, serum, and bronchoalveolar lavage fluid. Metabolomics Standard Initiative (MSI) Level 1: metabolites identified by the matches of MS/MS spectra and LC retention time with those of chemical reference standards acquired on the same analytical platform**.** MSI Level 2: metabolites identified by the similarities of MS/MS spectra and physicochemical properties with public/commercial spectrum libraries. MSI Level 3: metabolites putatively identified based on physicochemical characteristics and spectrum similarity to known compounds of a chemical class.

Table S2. Measured values for the features in lung, serum, and bronchoalveolar lavage fluid.

Figure S1. PCA score plots of PR8-infected mice sera, lung tissues and bronchoalveolar lavage fluid.

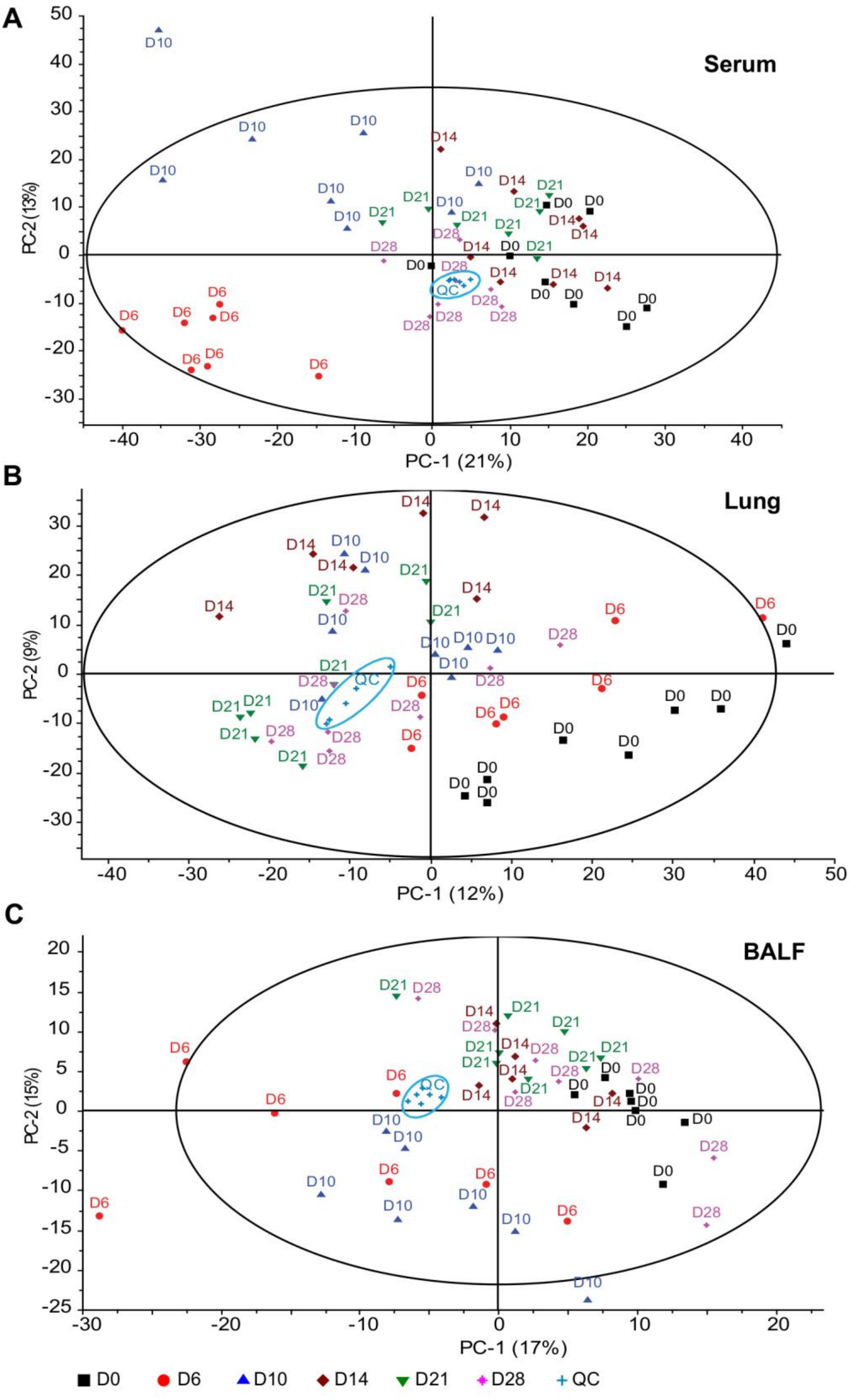

Figure S2. Identification of cyclic adenosine monophosphate (cAMP) as a differential metabolite in the lung.

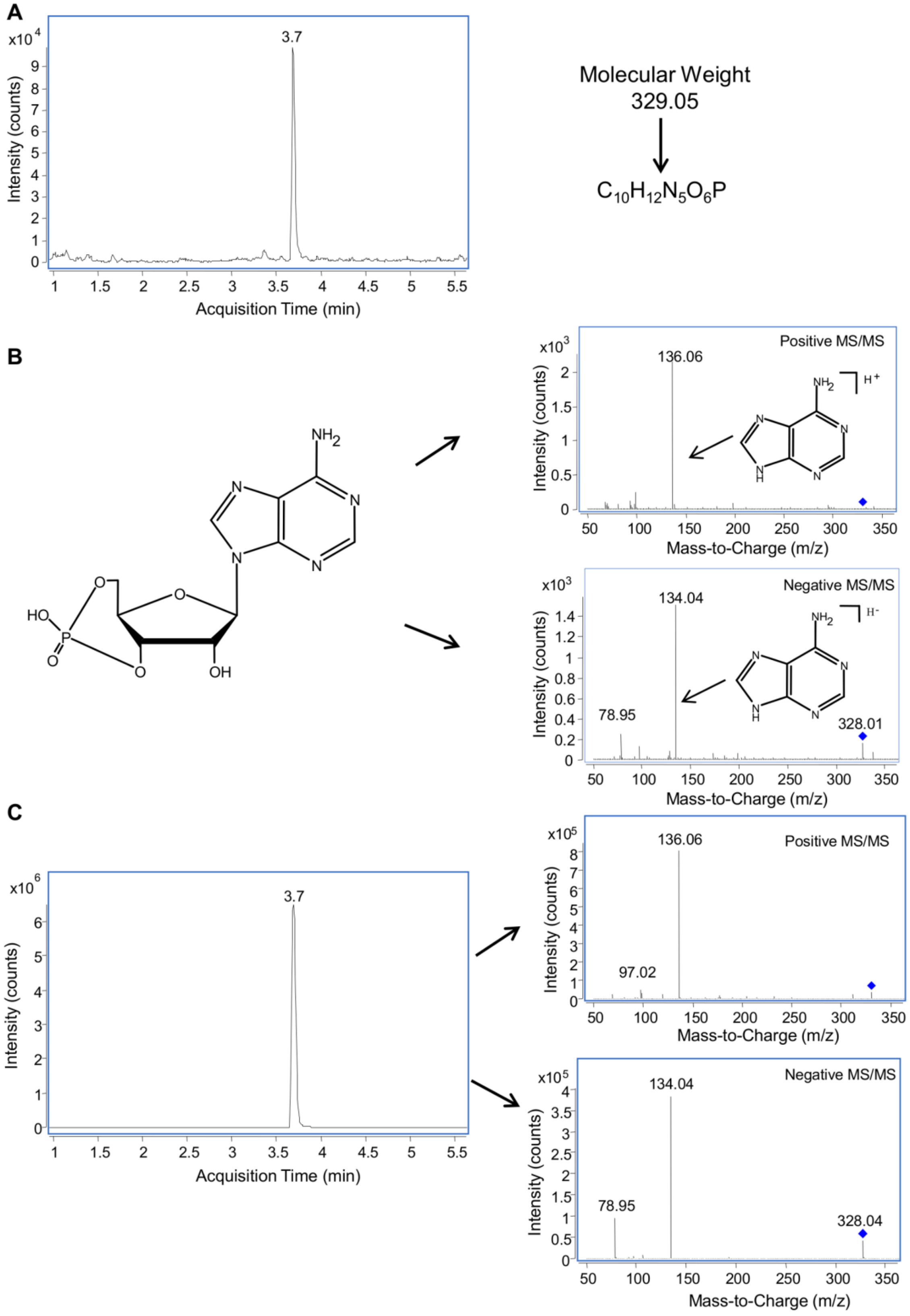

Figure S3. Pathways analysis with Ingenuity Pathway Analysis and Metaboanalyst based on differential metabolites in the lung.

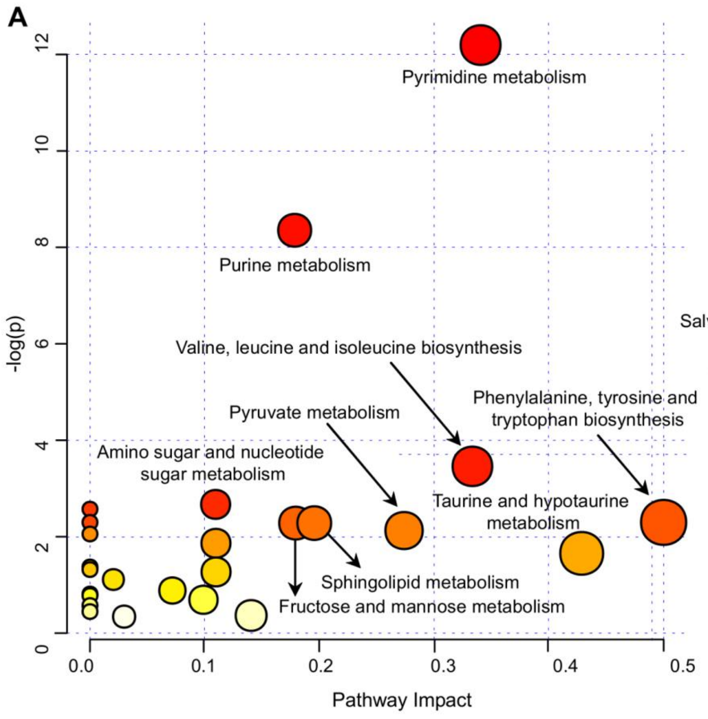

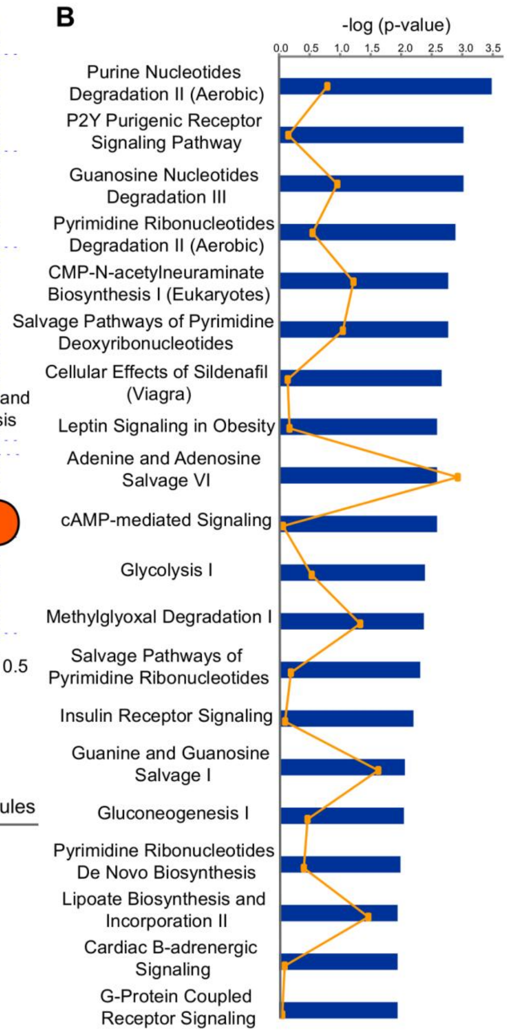

C

Name	p-value	# Molecules
Cellular Growth and Proliferation	2.66E-06 - 4.66E-02	17
Cellular Development	2.74E-04 - 4.66E-02	12
Free Radical Scavenging	4.01E-04 - 4.24E-02	9
DNA Replication, Recombination and Repair	9.52E-04 - 2.14E-02	9
Molecular Transport	9.52E-04 - 4.79E-02	12

Figure S4. Pathways analysis with Ingenuity Pathway Analysis and Metaboanalyst based on differential metabolites in serum.

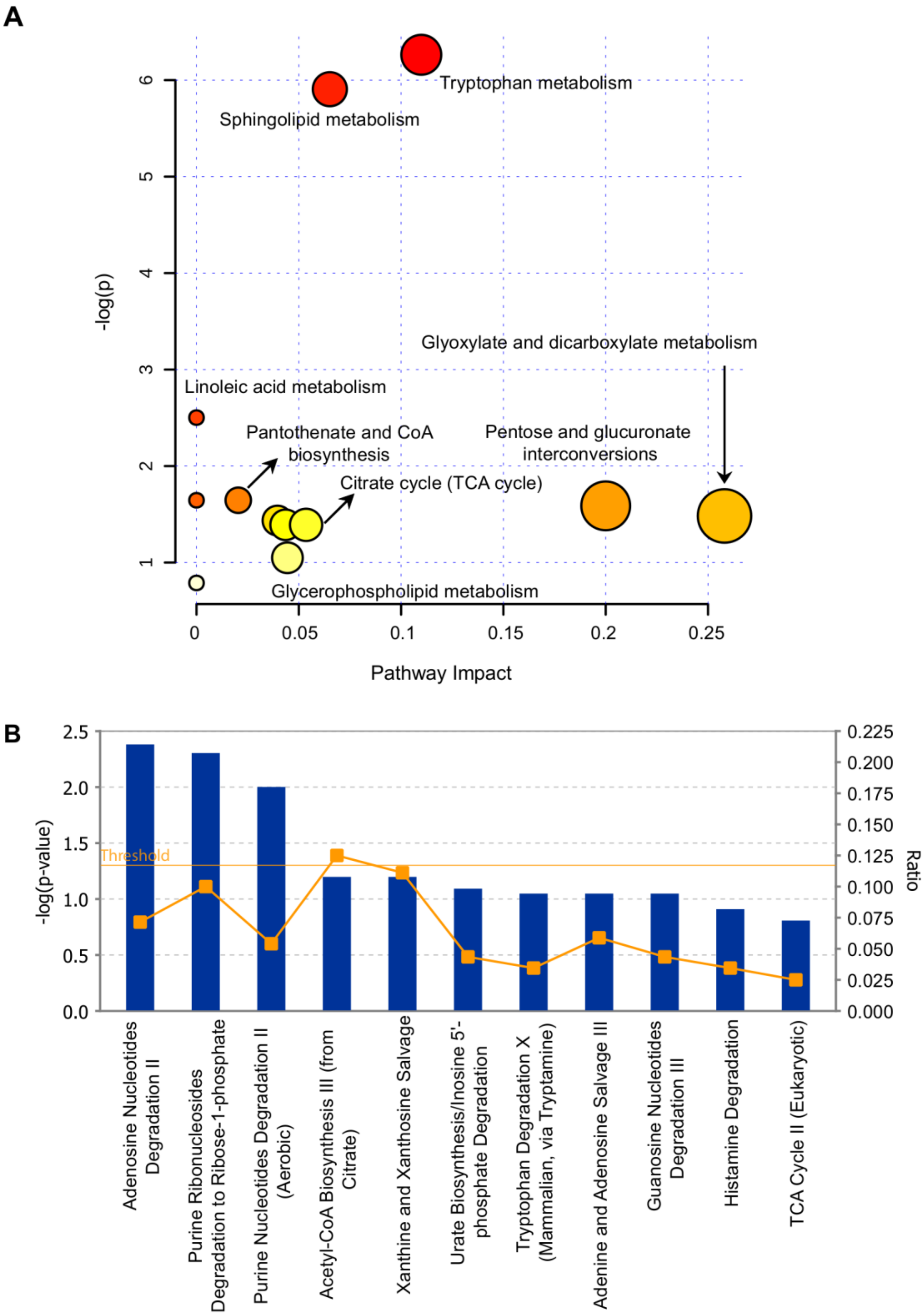

Figure S5. Pathways analysis with IPA and Metaboanalyst based on differential metabolites in BALF.

A

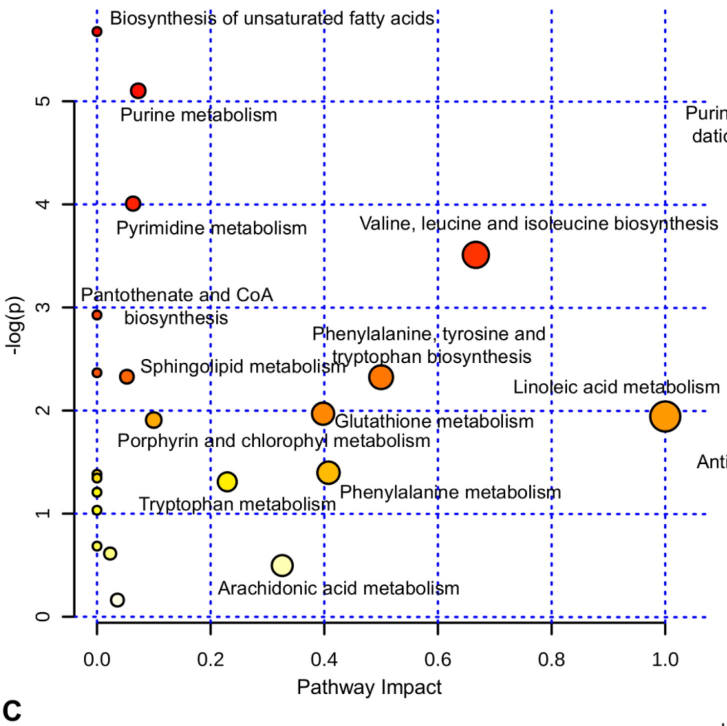

B

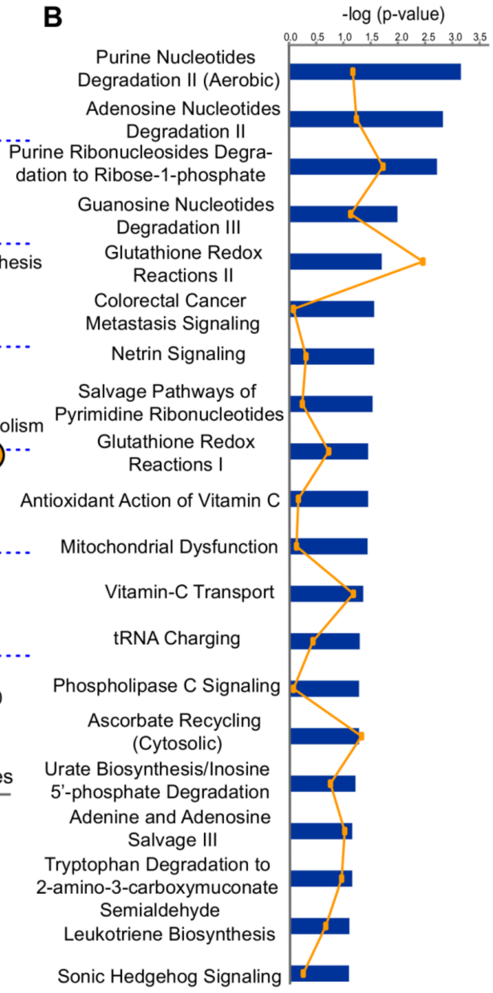

C

Name	p-value	# Molecules
Cellular Growth and Proliferation	6.64E-09 - 2.09E-02	21
Cellular Development	1.22E-08 - 3.20E-02	17
Free Radical Scavenging	1.39E-08 - 4.08E-02	17
Cell Death and Survival	9.59E-07 - 3.80E-02	19
Cellular Assembly and Organization	7.80E-06 - 3.20E-02	12

Figure S6. Restoration of surfactant phospholipid synthesis by perlipin A.

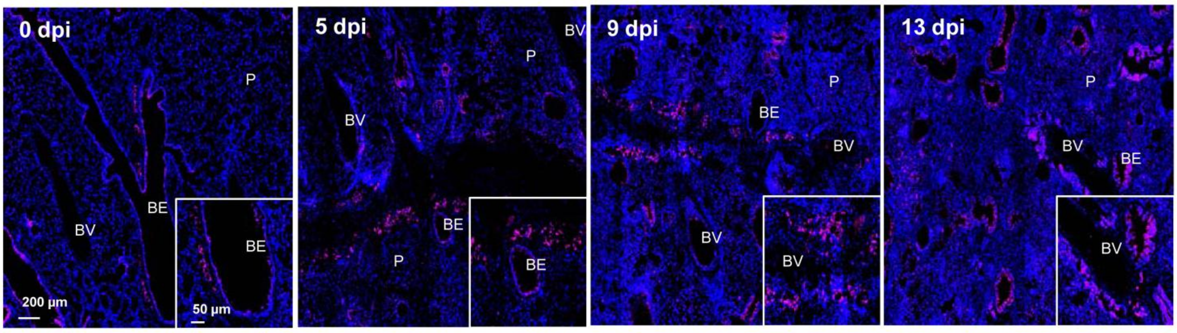

Supplement: Supplementary Information [file srep26076-s1.pdf]
